# Autonomous mobile clinics

**DOI:** 10.2471/BLT.22.288985

**Published:** 2022-09-01

**Authors:** Shaoshan Liu, Ao Kong, Yuzhang Huang, Xue Liu

**Affiliations:** a BeyonCa, No. 8, Ba He Bei An, Chaoyang District, Beijing,100016, China.; b United Nations Technology Bank for Least Developed Countries, Gebze, Türkiye.; c Johns Hopkins University Bloomberg School of Public Health, Baltimore, Maryland, United States of America.; d School of Computer Science, McGill University, Montreal, Canada.

Autonomous mobile clinics, currently in prototype form, are self-driving vehicles equipped with medical diagnostic equipment, telemedicine capability and an artificial intelligence (AI) software that can perform some of the tasks of health professionals, such as disease screening and basic diagnostics. Such clinics have the potential to revolutionize health-care delivery by bringing health-care services to hard-to-reach populations.^1^ Because they are autonomous, these clinics can independently navigate to places where health-care services are needed, while their mobility allows health-care access on-site. These clinics are equipped with conversational and visual user interfaces to facilitate their use by all population groups. In addition, the AI software incorporated into these clinics can serve an unlimited number of patients, and only cases that cannot be handled by the software would require the intervention of remote doctors, thus potentially reducing the cost for the human workforce.^2^

Each clinic contains several technologies, including traditional telemedicine, medical equipment integration, electronic health records, AI software and autonomous driving.^3^ The telemedicine^4^ component consists of two types of data communication: (i) transmission of raw sensing data (that is, collected from biomedical sensors and medical equipment) and (ii) video streaming, which allows interactive communications between patients and remote doctors.

Various types of on-vehicle medical equipment perform point-of-care testing, such as ultrasound and medical imaging equipment. However, until now, medical equipment manufacturers have enforced their own data standards – in structure, format and layout – preventing a seamless health-care delivery experience. Hence, a standard method of integrating and interpreting the enormous amount of sensing data is required.

The autonomous mobile clinics use electronic health records to collect and store patient data gathered by the equipment on board, such as heart rate, blood pressure and oxygen saturation, among others. These records enable integrated care through the AI software or telemedicine. Combined with valuable diagnostic data from remote doctors, these records are essential for improving the AI software, since the effective machine learning algorithm training is often constrained by the availability of integrated health records. For instance, screening for some diseases, such as dengue, can be facilitated by AI software.^5^

During the coronavirus disease 2019 (COVID-19) outbreak in China, researchers demonstrated the feasibility of using self-driving vehicles for contactless goods transportation.^6^

The ability of autonomous mobile clinics to provide accessible and affordable care will contribute to achieving universal health coverage (UHC), an objective that is high on the political agenda.^7^ Inequitable access to health services within and among countries as well as financial hardships associated with health services continue to impede progress towards achieving UHC.^7^ Taking note of the potential of autonomous mobile clinics to address these issues, the United Nations Technology Bank for Least Developed Countries^8^ consulted with industry and academic partners about possible approaches to pilot autonomous mobile clinics in one or two low-income countries, later expanding to more such countries.^9^

The pilot, still in its planning phase, will use a three-pronged approach: (i) screening for endemic diseases by using AI software, with specific algorithms for each type of disease; (ii) training autonomous vehicles to navigate remote and rural landscapes; and (iii) developing context-relevant solutions with local communities and health workers to form a health-care delivery package that connects the in-vehicle diagnosis, patients’ medical records, and referrals to telemedicine or in-person care as the next step, when necessary.

In addition to providing accessible and affordable health care, autonomous mobile clinics have potential for health-care response during crises, such as infectious disease outbreaks or disasters. For instance, autonomous vehicles have been used in emergency medical services, such as transporting COVID-19 patients to prevent exposure of emergency medical service workers to high coronavirus infection risks.^10^ During an outbreak, autonomous mobile clinics could independently navigate through the affected area, screening people with symptoms, and providing suitable treatment. These functions can be implemented with AI technologies.

We believe that reaching scalable outcomes is possible, using autonomous mobile clinics to provide primary care to people who currently do not have access to health care. By connecting patients to health-care providers across the world through telemedicine and using AI software to improve health-care efficiency, inequities within health-care delivery can be reduced.
